# Normalizing a hyperactive mTOR initiates muscle growth during obesity

**DOI:** 10.18632/aging.100290

**Published:** 2011-02-28

**Authors:** David L. Williamson, Joshua C. Drake

**Affiliations:** Department of Exercise and Nutrition Sciences, School of Public Health, University at Buffalo, Buffalo, NY 14214, USA

Functional impairment is a major concern in the obese population, leading to reductions in everyday activities [1]. Obesity-related reductions in muscle function are due to a loss of muscle mass (i.e. sarcopenia), which occurs largely from an imbalance between the rates of protein synthesis and degradation [2,3]. A major controlling mechanism for muscle peptide/protein formation is messenger RNA (mRNA) translation. Initiation of translation is regulated by hormones and the diet through alterations in the mammalian Target of Rapamycin (mTOR) [4,5,6]. Despite hyperactivation of the growth-promoting, nutrient-sensing mTOR pathway, atrophy persists in obese muscle [7]. Chronic hyperactivation of mTOR signaling is atypical outside of disease states, such as obesity, dyslipidemia, hypercholesterolemia, or certain types of cancer. With the obesity rate growing at an alarming rate, there is a critical need to determine how obesity-related sarcopenia can be limited, since metabolic homeostasis is positively linked to muscle mass.

Given the hyperactive state of mTOR signaling in obese skeletal muscle, normalizing mTOR signaling (to levels observed in lean mice) may be one avenue of limiting the resultant sarcopenia. However, treatment of obese rodent models with the mTOR inhibitor, rapamycin, showed limited improvement in insulin sensitivity, despite reductions in adiposity [8]. Although, recent studies show that aged [9] and cancer prone [10] mice can withstand chronic rapamycin treatment. This suggests that the efficacy of chronic rapamycin treatment may rely upon the dose, delivery method, tissue-specific effects, and length of treatment. This issue requires more work. Likewise, the use of insulin sensitizers and/or AMP-activated protein kinase (AMPK)-agonists [11] has proven beneficial in improving obesity-related metabolic complications in skeletal muscle. AMPK is a well-characterized sensor of the cell's energy status [12]. Compounds like AICAR (an AMP mimetic) and metformin, lead to the activation and phosphorylation of AMPK. AMPK activation promotes enhanced expression of skeletal muscle oxidative-related enzymes, proteins, and metabolism, which are consistent with the findings that obese skeletal muscles are less oxidative and have lower AMPK activation (during fasted conditions). At the same time, AMPK activation also inhibits mTOR signaling [13,14]. However, it seems counterintuitive to inhibit an important growth-mediated pathway (i.e. mTOR), regulating muscle mass, so that skeletal muscles can grow.

Our recent data [15] show that short-term (2-week), daily treatment of obese (ob/ob) mice with AICAR normalized their hyperactive, fasted-state mTOR signaling. Along with the expected reductions in circulating blood glucose and insulin concentrations, and muscle lipid and glycogen content after AICAR treatment, translational capacity and mass (including muscle fiber areas) of the plantar flexor muscle complex were significantly increased in the obese treated mice. It is our view that the oxidative metabolism/capacity of the muscle and the regulatory processes of muscle growth (i.e. mTOR and translational control) need to be normalized to elicit growth in insulin resistant (e.g. obese, aged) muscle. There are emerging data [16,17,18,19,20] that support our contention. Thus, gaining control of these initial signals and processes in obese, insulin resistant, and/or aging skeletal muscle with mTOR antagonists (e.g. rapamycin, metformin), may be beneficial to limiting sarcopenia and sarcopenia-related dysfunction.
